# Dental color measurement to estimate age in adults: a systematic review and meta-analysis

**DOI:** 10.1007/s12024-024-00798-4

**Published:** 2024-03-08

**Authors:** Jaime Martín-Martín, Ignacio Santos, María J. Gaitán-Arroyo, Juan Suarez, Leticia Rubio, Stella Martín-de-las-Heras

**Affiliations:** 1 Area of Legal and Forensic Medicine. Department of Human Anatomy, Legal Medicine and History of Science, University of Malaga, Bulevar Louis Pasteur 32, 29071 Malaga, Spain; 2 Biomedical Research Institute of Malaga (IBIMA), Malaga, Spain

**Keywords:** Age estimation, Teeth, Color, Forensic odontology, Forensic anthropology, Legal medicine

## Abstract

Age estimation is a major challenge in anthropology and forensic odontology laboratories, as well as in judicial settings, as one of the tools used in human identification. The aim of this study was to evaluate the usefulness of age estimation methods based on the accurate measurement of tooth color changes. A systematic review was carried out following the recommendations of the Preferred Reporting Items for Systematic Reviews and Meta-Analyses and in compliance with Cochrane criteria recommendations (PROSPERO registration number CRD 42022343371). An electronic search was performed in the following databases: Pubmed, Web of Science, Medline, Current Contents Connect, SciELO, KCI-Korean Journal Database, Derwent Innovations Index and Russian Citation Index. The search strategy yielded a total of 18 articles. A randomized meta-analysis model of the results for the CIE L*a*b* color variables stratified by age (less than 30 years, 30-60 years, 60 years and older) was performed with 9 of the 18 studies included in this systematic review. According to our results, sex and location of color measurement are the most influential factors in color estimation. All studies were carried out in healthy anterior teeth by spectrophotometry as the most commonly used method for color measurement, with CIE L*a*b* being the most commonly analyzed parameters. Studies based on age as a dependent variable showed R^2^ values between 0.28 and 0.56, being higher in ex vivo teeth. Studies based on age as an independent variable showed R^2^ values ranging from 0.10 to 0.48. The random model showed high heterogeneity for the L*, a* and b* parameters in all age groups, which is explained by discrepancies in age range and non-standardized conditions for color measurement. This systematic review highlights the need to protocolize age estimation studies that measure tooth color, in order to apply this method in different forensic settings.

## Introduction

Age estimation in living or dead individuals is a major challenge in anthropology and forensic odontology. Age estimation not only assists in criminal or civil proceedings involving minors or undocumented adults, but also facilitates the creation of a biological profile when identification is mandatory [1].

Teeth have been proved to be an important source for age estimation in both living and deceased individuals. Once tooth development is complete, degenerative or postformation dental changes are the appropriate markers for estimating age in adults [2]. The aging process in teeth is influenced by numerous external and internal factors (lifestyle, nutrition, type of work [3], toxic habits, diseases and treatments, among others) [4]. Common conditions and treatments such as teeth bleaching, orthodontics, trauma, prosthetic crowns, etc. may also alter the color of the tooth [5].

Thus, age-related dental changes can be modified by these factors, making the discrepancy between chronological and biological age greater and less accurate in adulthood [1], and hindering adult age estimation in the field of anthropology and forensic dentistry.

Several methods are available for age assessment in teeth. Biochemical techniques are applied to determine the age of an individual, such as aspartic acid racemization [6], methylation and telomere length [7–9]. However, biochemical studies are complex and involve tooth destruction [3], thus these methods are not the first choice in practical cases.

The most commonly used procedures are based on the study of morphological changes in dental structures caused by aging [1], such as secondary dentin apposition, root translucency, cementum apposition, attrition and dental color, among others. In this regard, panoramic dental radiographs have been used to estimate the age of adolescents and adults for forensic purposes. Apposition of secondary dentin is an important parameter for estimating age, as it is a continuous process throughout life that can be measured indirectly by the reduction of pulp area on radiographs [10, 11]. Other morphological techniques, such as microscopic techniques, oral cytology [12], computed tomography and magnetic resonance imaging [13], have also been applied to determine age. However, these techniques have the disadvantages of radiation exposure or destruction of teeth.

Changes in tooth color have been shown to contribute significantly to age estimation based on dental morphological changes [3]. The colors of enamel, dentin and cementum change with chronological age [14, 15]. Age-related changes in enamel color are due to surface cracking and increased nitrogen content, resulting in changes in light refraction. Changes in dentin color are attributable to changes in mineral and organic composition. The root color is less frequently affected than that of the crown, which is more exposed to external influences. [2]. These changes make the enamel thinner and yellower in older individuals (>45 years) compared to younger individuals [16]. In addition, a decrease in tooth brightness (L*) [17, 18] and an increase in redness (a*) with age have been reported [18].

Tooth color can be determined by visual comparison with a known standard (dental shade guide), although the ability to distinguish colors varies among observers or when biases are avoided by means of electronic color measuring devices, such as colorimeters, spectrophotometers, or digital cameras, among others [19]. The measurement of tooth color has great advantages for age estimation, since it does not involve the destruction of teeth, but it may be also very useful in living individuals, especially in countries that consider even low-dose exposure to radiological imaging to be inappropriate for age estimation. Several studies have compared different methods of age estimation [20–24], although a meta-analysis comparing the validation and best methods of age estimation using tooth color has not been performed to date. Therefore, the aim of this study was to evaluate the usefulness of age estimation methods based on accurate measurement of tooth color changes.

## Material and methods

This systematic review was conducted following the recommendations of the Preferred Reporting Items for Systematic Reviews and Meta-Analyses (PRISMA) statement [25] and in compliance with the Cochrane criteria. The protocol and the PI(E)COS question and the main searching strategy of this systematic review was registered in the International Prospective Register of Systematic Reviews (CRD42022343371).

### Selection criteria

The criteria for article selection were: descriptive studies, in vivo and ex vivo dental samples, human samples, color measurements as outcome variables, and relationship of tooth color variables with age. Systematic reviews, narrative reviews, case reports and studies with subjective color measurement systems or based on color guides were excluded.

### Databases searched

The bibliographic reference repositories used were Web of Science and Pubmed. All available databases were activated in the Web of Science search engine: Web of Science Core Collection, Medline, Current Contents Connect, SciELO Citation Index, KCI-Korean Journal Database, Derwent Innovations Index, and Russian Citation Index.

### Search strategy

The original search strategy was “colorimeter*”, “technique measures color”, “color measurement”, “shade*” combined with the term “teeth”. The term “age” or related was not included in the initial search strategy to avoid the risk of bias due to search complexity. This term was subsequently included in the Rayyan manager [26]. No linguistic or temporal limits were set.

### Selection process

The results obtained in the search were exported to the Rayyan systematic review manager [26]. In the Rayyan manager, the term "age" was included in the automated search tools as a criterion, and the studies obtained were extracted for the selection process. Subsequently, the duplicate detection tool was used. Two independent reviewers performed the initial selection process based on the title and the abstract of the results obtained. Then, the articles were read completely to determine their inclusion based on the previously established criteria. A third reviewer was involved in discordant decisions, and discussion ensued to reach consensus.

### Data collection process

The included articles were screened by two independent reviewers for manual extraction of the data of interest from the studies; no automation procedures were used. The data obtained were compared among the reviewers to reach consensus.

### Data Items

The following descriptive data were extracted from the articles included: author, year, origin of the sample, type of tooth, device used for the analysis of the sample, and analysis protocol used (setting device, measurement conditions, analysis system and location of the color measurement). Likewise, age estimation models based on the color variable and those that applied age to estimate color were also extracted from the studies in which they were developed.

### Study risk of bias assessment

According to the established inclusion criteria and the requirements of the present systematic review, the risk of bias could be identified in the following factors: incomplete protocol detailed, missing outcome data (lack of mean or standard deviation values), measurement of the outcome (use of non-comparable protocol), and selection bias (comparability of participants due to a lack of stratification by sample age). The risk detection process was performed manually and independently by two reviewers.

### Effect measures

The outcome variables extracted were means and standard deviations of the color systems, and the reliability parameters of the statistical models (r, R^2^, standard error estimate, area under the curve, sensitivity and specificity).

### Synthesis methods

The data extracted from the included studies were structured in five descriptive tables, three meta-analysis figures and an appendix to describe the protocol of the studies. Table 1 describes general aspects of the studies: author, year, country, type of tooth and type of sample. Table 2 shows outcome results: mean and standard deviation of the color variables for the age groups established by authors. Table 3 shows linear regression models with age as the dependent variable and Table 4 shows receiver operating characteristics. Table 5 shows linear regression models to estimate the color with age as an independent variable. A random meta-analysis model of the results for the CIE L*a*b* color variables stratified by age groups (under 30, 30-60, 60 and older) was performed with the Metafor plugin based on the R-software package [27]. The random model assumed that there was a τ2 variability (in addition to sampling variability), which explains differences between studies.

**Table 1:** Summary of articles identified in the systematic literature review

Author	Country	Tooth	Sample type
Cho et al. [44]	Republic of Korea	Maxillary and mandibular anterior teeth	In vivo
da Silva et al. [42]	Brazil	Right or left maxillary central incisor	In vivo
Demirel and Tuncdemir [5]	Turkey	Maxillary central incisors	In vivo
Devos et al. [40]	Belgium	Ex vivo and human skeleton In vivo: left or right maxillary central and lateral incisor and upper canine	Ex vivo and In vivo
Eiffler et al. [33]	Germany	Maxillary anterior teeth and first bicuspids. Mandibular canines and central incisors	In vivo
Gómez Polo et al. [29]	Spain	Maxillary central incisors	In vivo
Gozalo-Diaz et al. [43]	USA	Maxillary central incisors	In vivo
Greta et al. [39]	Romania	Central and lateral incisors, canines, premolars and molars.	In vivo (Non vital teeth and vital teeth as reference) *Whenever possible, the matching contralateral vital tooth was measured as a reference*
Haddad et al. [34]	Germany	The six upper and six lower anterior teeth	In vivo
Haralur [18]	Saudi Arabia	Maxillary central incisors	In vivo
Hassel et al. [35]	Germany	Maxillary central incisors	In vivo
Karaman et al. [36]	Turkey	Right maxillary central and lateral incisors and canine teeth	In vivo
Kim [37]	Republic of Korea	Maxillary central incisors	In vivo
Krasniqi et al. [41]	Kosovo	Left maxillary central and lateral incisors and canines	In vivo
Martin-de las Heras et al. [31]	Spain	Healthy erupted human permanent teeth (molars excluded)	Ex vivo Human skeletal remains
Martin-de-las-Heras et al. [32]	Spain	Maxillary central incisors and canines	In vivo
Rubiño et al. [30]	Spain	Right maxillary central incisor	In vivo
Wee et al. [38]	USA	Maxillary anterior teeth	In vivo

In vivo: not extracted tooth, Ex vivo: tooth extracted

**Table 2:** Age estimation based on the CIE system

Author	Age (yrs)	Nº teeth and sex	CIE L* a* b*;CIE L*C*H*; CIE XYZ							
			L	a	b	C	H	x	y	z
Cho et al. [44]	29±6.8	8 men 39 women	74±3.4	5±1.5	19.4±4.0	20.1±4.2	75.5±3.1			
da Silva et al. [42]	7-12	25 men 41 women	50.2±2.6	33.1±4.7	-74±4.4					
	13-20		50.2±2.3^*^	33.2±4.7	-73.9±3.8					
	21-30		56.1±5.1^*^	23.6±7.8^*^	-65.7±6^*^					
	31-40		52.9±3.9	28±6.1^*^	-67.9±4.4^*^					
	41-50		50.2±4.7	30.3±3	-70.8±3.2					
	51-64		45.3±4.1	31.2±6.4	-69.8±4.1					
Demirel and Tuncdemir [5]	˂35	177	78±5.32^#^	0.29±1.29^*^	17.48±4.87^8^					
	35-55	102	75.15± 5.9	0.68±1.17	20.58±8.16					
	˃55	23	72.33±6.37	1.35±1.77^*^	22.7±7.58^*^					
Eiffler et al. [33]	54-66	122	77.1±5.9			23.3±4.4	91.8±3.3			
	73-75	75	67.1±5.7			23.9±6.1	88.4±4.4			
Gómez Polo et al. [29]	16-89	671 men	74.98	0.59	21.42	21.53	89.90			
		690 women	77.51^*^	-0.32	18.31	18.37	91.87			
Gozalo-Diaz et al. [43]	18-85	120	77.3±6.5	4.2±2.1	10.5±4.2					
Greta et al. [39]	17-70	286 (88 men; 130 women)	86.2±5.18	-0.98±1.51	18.87±5.23	18.88±5.2	93.96±4.23			
Haddad et al. [34]	14-82	2067 (60 men; 114 women)	55.3-95.9	-3.44-7.67	3.59-37.92					
Haralur [18]	18-29	75	80.26 ±2.64^*^	0.614±0.71	19.19±3.92					
	30-50	75	79.67±2.68	0.68±0.7	19.76±3.28					
	50-70	75	76.66±33.43	1.76±1.45	22.72±4.33					
Hassel et al. [35]	53-55	84	76.2±6.1	-0.3±2.2	23.8±4.5^*^	23.9±4.6^*^	91.4±4.2^*^			
	73-75	22	68.5±6.6	0.4±2.1^*^	21.3±5.3	21.4±5.4	89.8±4.8			
Karaman et al. [36]	15-70	202	1.98±3.16	1.61±2.17	-0.03±0.1					
Kim [37]	16-30	100 men	79.33±4.58	-0.26±0.21	19.16±4.36^*^					
	16-30	100 women	80.15±4.96	-0.42±1.49	17.05±3.43					
	31-59	137 men	76.09±4.99	0.21±1.3^*^	19.8±4.21^*^					
	31-59	137 women	78.14±4.77^#^	-0.41±1.28	17.32±3.62					
	60-89	100 men	71.52±4.78	1.49±1.32^*^	25.39±5.63^*^					
	60-89	100 women	72.77±5.66	0.82±1.35	21.19±4.35					
Krasniqi et al. [41]	20-29	882 teeth (98 subjects)	83.2^#^	-0.7	21.7					
	30-39	693 teeth (77 subjects)	81.5^#^	-0.7	21.6					
	40-49	720 teeth (80 subjects)	79.4^#^	-0.5	23.9					
Martin-de las Heras et al. [31]	10-89	135 men						0.389±0.012	0.412±0.008	0.197±0.019
	10-89	115 women						0.385±0.011^***^	0.410±0.008	0.203±0.019^*^
Martin-de-las-Heras et al. [32]	20–29	14	59.25±8.17	0.38±3.08	6.88±2.39			0.331±0.011	0.347±0.004	0.321±0.013^*^
	30–39	14	56.12±7.99	3.38±4.37	8.67±2.15			0.343±0.123	0.349±0.006	0.306±0.012
	40–49	16	56.67±6.11	1.27±1.96	8.48±2.23			0.338±0.009	0.351±0.005	0.309±0.013
	50–59	16	57.51±5.36	2.29±2.61	10.92±2.56			0.346±0.013	0.357±0.008^#^	0.296±0.011
	60–69	13	58.19±5.72	2.87±4.33	8.86±3.37			0.342±0.015	0.350±0.008	0.307±0.019
	≥70	15	57.21±4.41	4.04±3.29^*^	11.41±1.74^*^			0.352±0.008^*^	0.356±0.006	0.291±0.009
(Rubiño et al. [30])	15-50	600	67.6 (DT 7)	4.3 (DT 2.1)	12.1 (DT 3.3)					
(Wee et al. [38])	18-85	120	73.5±7.6	2.2±1.8	11.9±4.0					

*L *lightness, *C *chroma, *H *hue, *SD* standard deviation

*p <0.05; **p <0.01; ***p <0.001

**Table 3:** Age estimation based on equation models with age as dependent variable

Author	Analysis model	Age equation model		Reliability		
				r	R^2^	SE
Devos et al. [40]	Ex vivo Linear Regression model Male including location	Age = 165.79 – 2.06 L – 4.95 a + 1.04 b + 38.90 CV + 2.89 RM	CV: crown vestibular; RM: root mesial; RV: root vestibular: include either 1 or 0 into the model according to specific location. Sex (G) G=0 for male and G=1 for female. For the parameter tooth (T), substitute the numerical value of the specific one. The other tooth parameters become zero. In the case of tooth 13, all tooth parameters become zero.	0.7	0.49	
	Ex vivo Linear Regression model Male including location and sex	Age = 162.08 –2.15 L – 5.05 a + 1.43 b + 43.60 CV + 6.07 G		0.7	0.49	
	Ex vivo Linear Regression model Female including location	Age = 166.01 – 2.23 L – 5.31 a + 1.67 b + 48.20 CV		0.69	0.48	
	Ex vivo Linear Regression model Female including location and sex	Age = 162.08 –2.15 L – 5.05 a + 1.43 b + 43.60 CV + 6.07 G		0.7	0.49	
	In vivo Linear Regression model Including interaction between tooth and L parameter.	Age = 247.58 – 3.34 L – 3.70 a + 1.07 b + 0.20 T11 +0.11 T12 + 0.35 T21 + 0.29 T22 + 0.12 T23		0.74	0.56	
Martin-de las Heras et al. [31]	Ex vivo Linear Regression for fresh extracted teeth	Age =-339.4+1016.8x	Y: luminance in cd/m^2^ $WIC={Y}_{rel}+800\left({X}_{n}-x\right)+1700({y}_{n}-y)$ $Z\%=\left(\frac{Z}{{Z}_{n}}\right)100$ $WI=4Z\%-3{Y}_{rel}$ $YI=100\left(\frac{1-0.847Z}{{Y}_{rel}}\right)$	0.67	0.44	13.2
		Age =-580.4+1544y		0.72	0.51	12.3
		Age =183-639.2z		0.71	0.50	12.6
		Age =107.9-0.345Y		0.53	0.28	15.2
		Age =19.3-0.44WIC		0.75	0.56	11.7
		Age =104+1.4Z%		0.72	0.51	12.4
		Age =24.9-0.64WI		0.55	0.30	14.9
		Age =58.5+1.02YI		0.71	0.50	12.5

*SE* standard error of estimate, *YI* yellowness index

**Table 4:** Receiver operating characteristic (ROC) curve analysis for age estimation with colorimetric variables (L*, a*, b*), chromaticity coordinates (x, y, z) or whiteness (WIC, Z%, WI) and yellowness (YI) indexes in upper incisors

Author	ROC parameter		Age					
			≥30	≥40	≥50	≥65	≥70	≥75
Martin-de-las-Heras et al. [32]	L*	AUC	0.6	0.5	0.5	0.5	0.5	0.5
		Youden	60.74	60.74	49.81	50.92	59.23	52.04
		Se/Sp	77/57	78/42	95/18	94/19	73/41	91/20
	a*	AUC	0.8	0.7	0.7	0.7	0.7	0.7
		Youden	0.36	0.92	0.27	0.94	0.78	2.69
		Se/Sp	87/71	66/67	90/38	78/50	93/46	64/74
	b*	AUC	0.8	0.7	0.8	0.7	0.8	0.8
		Youden	9.21	9.21	9.22	10.09	10.43	10.44
		Se/Sp	59/85	66/78	75/72	78/79	86/76	91/74
	x	AUC	0.8	0.7	0.7	0.7	0.8	0.8
		Youden	0.335	0.336	0.335	0.342	0.340	0.344
		Se/Sp	78/85	80/64	88/52	78/66	93/53	91/51
	y	AUC	0.7	0.7	0.7	0.7	0.7	0.7
		Youden	0.351	0.351	0.353	0.353	0.354	0.354
		Se/Sp	56/92	65/85	70/79	78/59	80/71	82/69
	z	AUC	0.8	0.7	0.8	0.7	0.8	0.8
		Youden	0.309	0.304	0.301	0.3	0.301	0.298
		Se/Sp	71/92	70/75	72/72	78/60	93/58	82/65
	WIC	AUC	0.8	0.8	0.8	0.7	0.8	0.8
		Youden	-34.28	-34.28	-37.33	-42.35	-38.02	-42.35
		Se/Sp	74/92	78/67	81/63	72/60	93/54	91/60
	Z%	AUC	0.7	0.6	0.6	0.6	0.6	0.6
		Youden	25.36	25.36	25.36	23.15	19.68	22.27
		Se/Sp	87/57	91/42	93/31	79/30	73/54	81/36
	WI	AUC	0.8	0.8	0.8	0.7	0.8	0.8
		Youden	5.92	4.41	4.40	2.76	2.73	2.76
		Se/Sp	75/92	71/75	79/68	78/67	86/67	91/65
	YI	AUC	0.8	0.8	0.8	0.7	0.8	0.8
		Youden	25.77	26.47	25.22	28.25	28.54	28.59
		Se/Sp	68/92	68/78	86/61	78/70	86/72	91/70

*AUC* area under the ROC curve, *Se* sensitivity, *Sp* Specificity

**Table 5:** Linear regression models for color estimations with age as independent variable

Author			Linear	R^2^	R^2 mean^
Gómez Polo et al., 2015	L*	Male	y=87.98-0.28*years	0.48	0.45
		Female	y=88.03-0.23*years	0.45	
	a*	Male	y=-2.22+0.06*years	0.25	0.21
		Female	y=-1.84+0.03*years	0.22	
	b*	Male	y=13.55+0.18*years	0.24	0.17
		Female	y=13.94+0.10*years	0.13	
	C*	Male	y=13.50+0.18*years	0.24	0.17
		Female	y=14.00+0.10*years	0.13	
	h*	Male	y=96.24-0.14*years	0.25	0.21
		Female	y=93.36-0.10*years	0.20	
Gozalo-Diaz et al., 2008	L*	Male	L*=86.5 - 0.22 * Age(yrs)		0.36
		Female	L*=86.5 - 0.22 * Age(yrs) + 2.0		
	a*	Male	a*=1.9 + 0.05 * Age(yrs)		0.16
		Female	a*=1.9 + 0.05 * Age(yrs)		
	b*	Male	b*=15.4 + 0.10 * Age(yrs)		0.21
		Female	b*=15.4 + 0.10 Age(yrs) - 1.6		
Kim, 2018 Three different values can be assigned for age variables in this equation: 1, young age group (16–30); 2, middle age group (31–59); and 3, elderly age group (60–89).	L*	Male	L* =83.506-3.905 (age group)	0.28	
		Female	L* =84.522-3.69 (age group)	0.23	
	a*	Male	a* = -1.302 + 0.875 (age group)	0.21	
		Female	a* = -1.292 + 0.621 (age group)	0.10	
	b*	Male	b* =15.041 + 3.114 (age group)	0.19	
		Female	b* =14.250 + 2.068 (age group)	0.14	

## Results

**Fig. 1:**
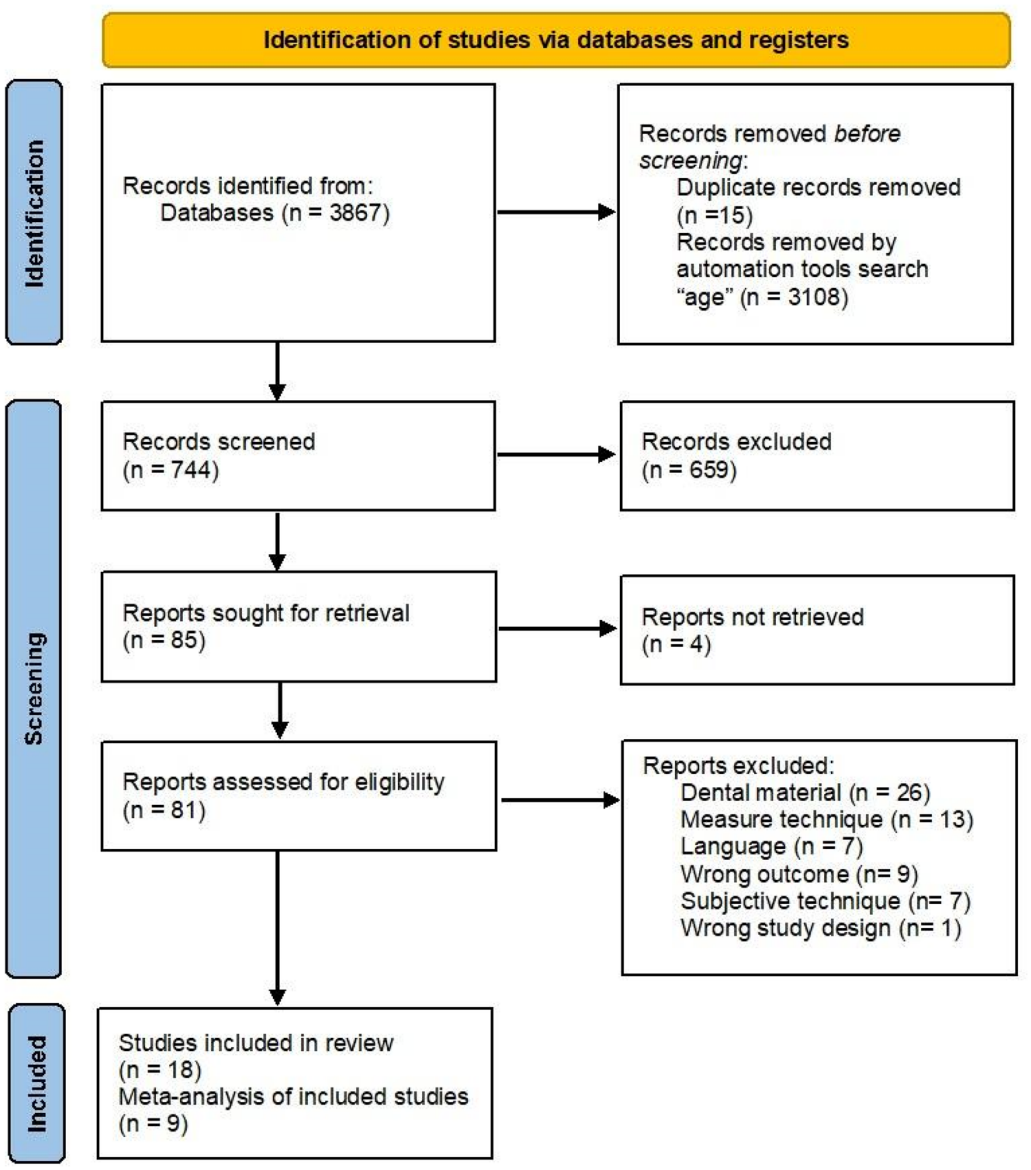
Flow diagram for the systematic review according to preferred reporting items for systematic reviews and meta-analyses (PRISMA guidelines)

Figure 1 shows the study selection process applied in the systematic review. The initial search strategy yielded 3867 results, of which 15 were duplicates and 3108 were discarded due to the automation process in the "age" search. A total of 744 studies were selected for title and abstract review, of which 659 were discarded following the inclusion and exclusion criteria. The remaining 85 were selected for full text review, 4 of which were not reported; thus, 81 reports were assessed for eligibility. The level of agreement observed between the two reviewers was 84% (k= .811 95%CI= .71; .905 p˂.05), indicating very good concordance [28]. Sixty-three studies were excluded after full-text reading and application of the established inclusion and exclusion criteria. Therefore, a total of 18 studies were included in the present systematic review (Fig. 1).

The risk of bias analysis was performed by two independent reviewers. The results showed a low level of risk related to design, detailed protocol and outcome data (mean and standard deviation). However, the lack of stratification of the results by age (selection bias) limited their inclusion in the meta-analysis. Although all the analyzed studies employed the same measurement system (CIE L*a*b* or CIE L*C*H*), the devices used were different. Therefore, the applicability of the obtained results could include small deviations in the final results.

The most common countries of the 18 studies were Spain (5 of 18) [17, 29–32] and Germany (3 of 18) [33–35] (Table 1). In eight of the included studies, it was not possible to identify the origin of the sample [5, 29, 30, 34–38]. All studies included in vivo upper anterior teeth for analysis, except one, which used ex vivo (non-vital) teeth [31]; the study by Greta et al. also analyzed non-vital teeth [39]. Five of the studies established smoking as an exclusion criterion [18, 29, 33, 40, 41]. However, the study by Kim et al. considered smokers among their inclusion criteria for a comparative analysis based on the color changes produced by smoking in teeth [37]. In general terms, the exclusion criteria defined by the authors were similar: restorations, aesthetic procedure (staining, fluorosis or whitening), attrition, gingival bleeding, caries, abraded lesion, cavities and stains [32, 42, 43]. Therefore, it can be considered that all the studies included in the present systematic review were carried out with healthy teeth (Table 1).

In eleven studies, color was measured by spectrophotometry [5, 18, 29, 32–37, 39, 41]. Spectroradiometry was used in three of the analyzed studies [32, 38, 43], colorimeters in three other studies [30, 40, 44], and a digital camera and a computer for color analysis in one of them [42]. Thirteen of the included studies used portable color estimation devices that could be used in reproducible environmental conditions (Appendix 1).

The environmental and lighting conditions for sampling in the different studies were natural (in clinics or laboratories), except for the use of ultraviolet light [42] and a cabinet or special chamber [31, 40]. Among the studies that carried out the color measurement using spectrophotometry, some of them adopted standardized lighting conditions [17, 36, 39], while in others the environmental conditions were not standardized [18, 32, 33]. In these cases, color measurement was performed by placing the probe tip in contact with the tooth surface, with most authors considering the middle third of the facial tooth surface to be the most appropriate region (Appendix 1). Particular environments were designed for spectroradiometry measurements according to the protocols established by different studies [32, 38, 43]. In these studies, the measurement was performed at a standardized distance of 8-9 centimeters from the measured object (Appendix 1).

The age of the sample, the number of teeth, the color system used (CIE L*a*b*, CIE LCH or CIE XYZ), and statistically significant differences observed by the different studies are shown in Table 2. Concerning the stratification of the analyzed samples, some studies segmented the results by age into three or more groups, which allowed obtaining comparable values and observing the change in tooth color between groups [5, 32, 37, 41, 42]; however, studies without age range groups made it impossible to establish this differentiation [17, 30, 31, 34, 36, 38, 39, 43].

All studies included both men and women. Four of the analyzed studies showed the results segmented by sex [17, 31, 36, 37]. The number of samples differed among the analyzed studies; the largest sample size was found in Krasniqui et al., with 2295 teeth [41], and the smallest in Cho et al., with 47 teeth [44] (Table 2).

Regarding the measurement system, fifteen of the included studies used the CIE L*a*b* system [5, 17, 18, 30, 32, 34–39, 41–44], four studies used the CIE L*C*H* system [17, 33, 35, 39], and two studies employed the CIE XYZ system [31, 32] (Table 2). The results of all the analyzed studies showed the color variables based on the mean and standard deviation, except for two studies: one that only provided mean values [17] and another based on means values and typical deviation [30].

Table 3 shows linear regression models with age as a dependent variable. The results of the analysis models could vary according to the sample (in vivo [intraoral measure] or ex vivo [extracted tooth]), the measurement location and sex. In the case of the study by Devos et al., the best model for estimating dental age was in vivo, with a value of R^2^=0.56; the standard error of age estimation was not reported [40]. According to the author's results, the proposed model underestimates the real age in the highest age categories and overestimates the chronological age in the lowest categories [40]. The linear regression model published by Martin de las Heras et al. (Age=19.3-0.44WIC) has a standard error of 11.7 years and r=0.75 for ex vivo fresh extracted teeth [31].

Table 4 shows the analysis of age as a dependent variable in Receiver Operating Characteristic (ROC) curve models. The different color variables were used to determine the sensitivity and specificity for estimating age in different age groups of ten years range (i.e., 20-29 years, 30-39 years, etc.). The Area Under the Curve (AUC) values ranged between 0.7 and 0.8 for all dental color parameters measured in the upper central incisors, except for the variables L* and Z%, with sensitivity values ranging between 56% and 93%, and specificity values between 38% and 92% [32].

Table 5 shows the regression models (equation) for estimating the tooth color variables, considering age as an independent variable in the model. The model with the highest predictive value was developed by Gomez Polo et al. (2015) [29]. The value of L* as dependent variable in regression models could be estimated with a reliability of 48.8% in men (y=87.98-0.28*years) and 45.1% in women (y=88.03-0.23*years) with a value of R^2^=0.48 and 0.45, respectively [29].

**Fig. 2:**
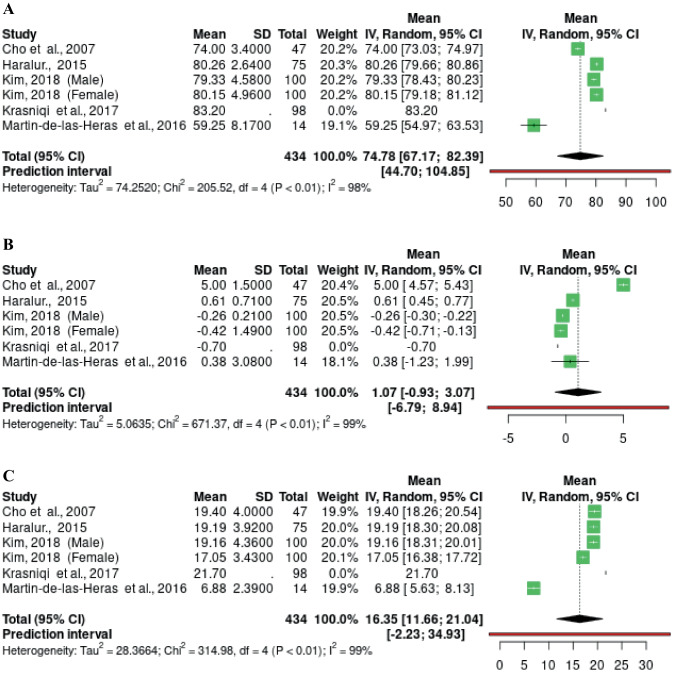
Forest plot of the meta-analysis performed on L* (**A**) a* (**B**) and b* (**C**) values in studies with individuals younger than 30 years old

**Fig. 3:**
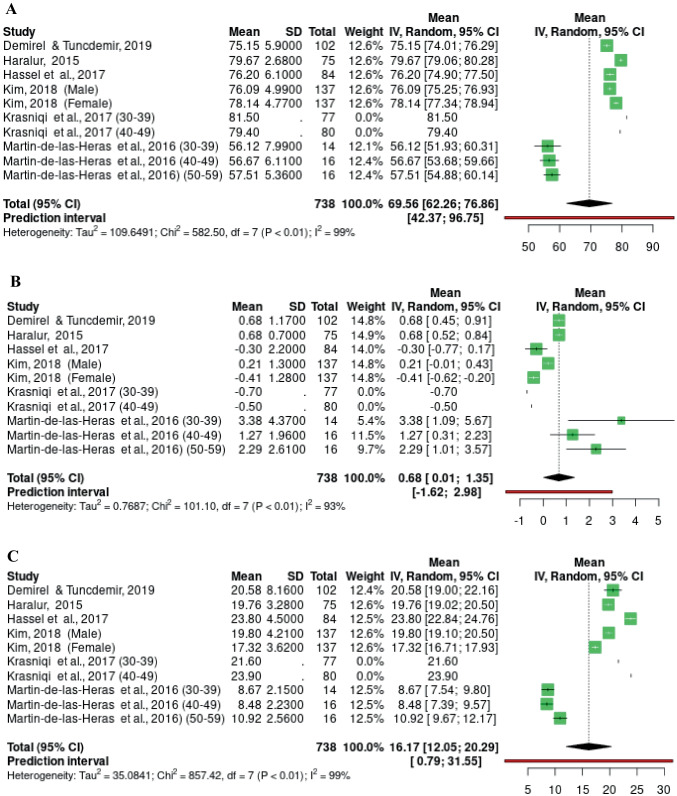
Forest plot of the meta-analysis performed on L* (**A**) a* (**B**) and b* (**C**) values in studies with individuals between 30-59 years old

**Fig. 4:**
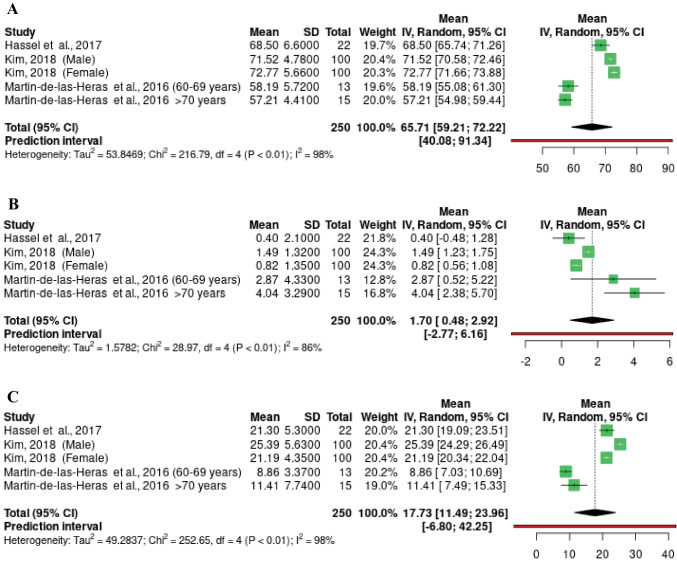
Forest plot of the meta-analysis performed on L* (**A**) a* (**B**) and b* (**C**) values in studies with individuals over 60 years old

Figures 2, 3 and 4 show the random-effects meta-analysis model performed on tooth color variables in the CIE L*a*b* system grouped by age: under 30 years (Fig. 2), 30-59 years (Fig. 3) and 60 years and older (Fig. 4). The study carried out by Da Silva et al. was not included in the meta-analysis, as it used non-comparable techniques (ultraviolet light) [42].

Seven groups of samples under 30 years of age from 6 studies were included (Fig. 2). In a total of 434 samples, heterogeneity (I^2^) was very high in all three analyses, thus the variability is not explainable by simple sampling error. The study by Kim contributed 200 samples (100 male and 100 female), representing 46.08% of the present analysis samples [37]. The L* mean value for this age group was 74.78 (95%CI 67.18; 82.39 *p* < 0.01) (Fig. 2A). The a* mean value was 1.07 (95%CI -0.93; 3.07* p* < 0.01) (Fig. 2B). Likewise, the b* mean was 16.35 (95%CI 11.66; 21.04* p* < 0.01) (Fig. 2C). For the 3 variables analyzed, heterogeneity was greater than 98%.

Ten groups of samples aged 30-59 years from 6 studies were included (Fig. 3). The study by Kim (2018) contributed 274 samples (137 male and 137 female), representing 38.05% of the analysis samples [37]. The L* mean value for this age range was 70.07 (95%CI 62.95 ; 77.18 *p* < 0.01) (Fig. 3A); the a* mean was 0.51 (95%CI -0.06; 1.09 *p* < 0.01) (Fig. 3B), and the b* mean was 16.17 (95%CI 12.05 ; 20.29 *p* < 0.01) (Fig. 3C). For the 3 variables analyzed, the heterogeneity of the results was very high.

Five groups of a total of 250 samples aged ≥60 years from 3 studies were included (Fig. 4). The study by Kim contributed 200 samples (100 male and 100 female), representing 80% of the analysis samples [37]. The L* mean value was 65.71 (95%CI 59.21; 72.22 p < 0.01) (Fig. 4A); the a* mean was 1.70 (95%CI 0.48; 2.92 p < 0.01); and the b* mean was 17.77 (95%CI 11.49; 23.96 p < 0.01) (Fig. 4C). Heterogeneity was 98% for the L* and b* values, and 86% for the a* values.

## Discussion

Age estimation is a major challenge in anthropology and forensic odontology laboratories, as well as in judicial settings, since it is one of the tools used in human identification. Color tooth measurement is a very valuable method for estimating age in living individuals, complying with medical ethical standards, especially in some countries where low-dose X-ray exposure is considered unethical for age estimation. This review provides a meta-analysis of methods for age estimation by accurate measurement of aging-related changes in tooth color.

According to the present review, the type of tooth used to estimate age based on color is healthy anterior teeth (incisors and canines) free of caries, cavities, attrition, endodontics, reconstruction, breakage, bleaching, or abnormal staining. Previous studies have analyzed tooth color measurement devices [19], mainly for clinical purposes, and concluded that spectrophotometers, colorimeters and imaging systems are useful and relevant tools. However, in our systematic review, the most commonly used instrument was the spectrophotometer; this may be mainly due to the fact that spectrophotometric shade analysis is accurate, reproducible and portable [45], which facilitates its transport in real forensic cases. Regarding the systems employed, we observed that fifteen of the included studies used the CIE L*a*b* system [5, 17, 18, 30, 32, 34–39, 41–44], four studies used the CIE L*C*H* system [17, 33, 35, 39], and two studies used the CIE L*a*b* system with parameters X,Y and Z [31, 32] (Table 3). According to our results, the CIE L*a*b* system has been the most widely used, probably due to its advantages, such as an easier interpretation of the psychophysical dimensions of color perceptions and the possibility to estimate the magnitude of the differences between two color stimuli using the chromaticity diagram [40].

The statistical methods proposed to estimate age according to tooth color in adults are linear regression models and ROC curves, with the former being the most commonly used. These studies used age as a dependent (Table 3) or independent (Table 5) variable. The studies based on age as the dependent variable showed R^2^ values between 0.28 and 0.56, being higher in cases of ex vivo teeth (Table 3). Among the studies on ex vivo teeth, the results of studies that estimated age from fresh extracted teeth [31] should be differentiated from the results of others that analyzed stored extracted teeth [40], since storage time may affect tooth color. However, the results obtained from the ex vivo studies are similar in Martin de las Heras and Devos [31, 40], although the correlation coefficients of the former were higher. In addition, different works show that ex vivo teeth are darker than in vivo teeth [15, 40]; therefore, age estimation using tooth color in corpses should be taken with caution [15].

Although the standard error of the models should be applied in practical forensic cases and compared with other populations, some studies did not reflect this [29, 37, 40, 43]. Studies based on age as an independent variable showed an R2 with values ranging from 0.10 to 0.48. In this regard, the model with the highest predictive capacity was the one developed by Gomez Polo et al., with L* representing the best predictive parameter in both males and females [29]. However, these results should be taken with caution, as age is not considered a covariate in these studies and there may be an important effect of age on data interpretation. Regarding the ROC curves, to our knowledge, only the study by Martin-de-las-Heras et al. performed the analysis using this method [32]. This study showed AUC values from 0.7 to 0.8 for all dental color parameters measured in upper central incisors, except for the variables L* and Z%, with sensitivity values ranging from 56% to 93% and specificity values ranging from 38% to 92% [32]. In this sense, a diagnostic test is considered “highly accurate” with an AUC value >0.9, “useful for some purposes” with a value of 0.7–0.9, and “poor” with a value of 0.5–0.7 [46]. Applying this statistical interpretation, the method developed by Martin-de-las-Heras et al. is considered useful for age estimation [32].

Age estimation based on tooth color has certain limitations. Tooth color may vary as a result of age, but also due to other causes such as smoking, tobacco mastication, poor dental hygiene, consumption of foods and beverages with chemical components (coffee, tea, red wine), and excessive use of fluoride, as well as some treatments, illness and genetic factors [41]. Thus, most studies determined tooth color in non-restored, non-discolored and non-smokers’ teeth, which could limit the extrapolation to real cases [5, 32, 41]

Several aspects may influence age estimation. The most relevant seem to be the location of the color measurement and sex [40]. In ex vivo teeth, it is recommended to perform the color measurements on the vestibular surface of the crown and on the mesial and vestibular surface of the root. In the case of in vivo teeth, it is recommended to take measurements on the vestibular enamel at least 2 mm coronal to the gingival border [32, 40]. On both types of teeth, it is recommended to place the device in full contact with the tooth surface and measure color variables several times (from 3 to 5) using the average of the measurements [32, 40]. Regarding sex, men and women participated in all studies, but there was no homogeneity in the sample. In addition, there were discrepancies among studies. For example, Gomez Polo et al. observed that the mean value of L* and H* was significantly higher in women than in men, while the mean value of a*, b* and C* was significantly higher in men [17]. A similar situation occurred in the study by Gozalo-Díaz et al., where it was observed that women had higher values of lightness and lower values of yellowness than men in the analyzed teeth [43]. Martín de las Heras et al. showed statistically significant differences between sexes, especially in the values of the X and Z parameters [31], and Demirel and Tuncdemir observed that women had lighter teeth than men [5]. In contrast, for Hassel et al., sex was not a significant factor [35].

A randomized meta-analysis model of the results was performed for the CIE L*a*b* color variables stratified by age. In order to pool the largest number of studies and samples and obtain higher forensic relevance in the meta-analysis, the age groups were classified as follows: under 30 years, between 30 and 60 years, and over 60 years. The random model assumed that there was a τ2 variability (in addition to sampling variability), which explains differences between studies. Regarding the group under 30 years of age, our results showed a high heterogeneity for the parameters L* (I^2^=98%), a* (I^2^=99%), and b* (I^2^=99%). The group between 30 and 60 years of age showed high variability for the parameters L* (I^2^=99%), a* (I^2^=93%), and b* (I^2^=99%). The group older than 60 years also showed heterogeneity for the parameters L* (I^2^=98%), a* (I^2^=86%), and b* (I^2^=98%). All analyses showed very high heterogeneity (I^2^). The percentage of heterogeneity in effect sizes cannot be attributed to sampling error [47] or sample size. The heterogeneity is explained by several factors, such as the wide age range of each of the studies and the non-standardized conditions for color measurement [48]. In this regard, the colorimeters used in each study were different (with different sensitivity and precision), the environments and lighting conditions of the measurements were not the same, and the measurements were aggregated by very wide age ranges, affecting both the mean values and the confidence intervals.

According to our results, we recommend the use of linear regression models with age as the dependent variable, calculating the standard error for age estimation. It is also recommended to standardize the experimental conditions with a color measurement protocol, increasing the sample size of the studies, and including small age ranges, both sexes and different ethnicities.

To the best of our knowledge, this is the first systematic review to analyze methods for estimating age based on accurate measurement of tooth color changes. However, our study has some limitations. The articles were grouped into different age groups (under 30 years, between 30 and 60 years, and over 60 years) in order to include the maximum number of research papers in the meta-analysis, excluding others. For this reason, the results should be taken with caution; however, the randomized meta-analysis model attempts to balance this. The lack of standardization among studies may also be a limitation to extract data not only in our research but also in any future studies. In this regard, an international color-age estimation protocol tool is crucial to ensure consistency of data extraction and comparison between studies. In addition, despite the meticulous search strategy used, some articles may have been overlooked in the present study.

In conclusion, the use of linear regression models with age as the dependent variable to calculate the standard error is recommended to estimate the interval of dental age. Color measurement should be based on an accurate and objective technique such as spectrophotometry, which is the most commonly used method, placing the device in full contact with the tooth surface and measuring the color variables several times using the mean of the measurements. It is recommended to increase the sample size of the studies, including small age ranges, equation models for different types of teeth, both sexes, and different ethnicities. This systematic review also highlights the need to protocolize age estimation studies that measure tooth color in order to apply this method in different forensic settings.

## Key points

1. This is the first systematic review and meta-analysis based on dental color estimation.
2. Linear regression models with standard deviation are recommended for age estimation.
3. The meta-analysis showed heterogeneity among the studies; several factors are discussed.
4. Small age ranges and standardized measurement conditions would improve age estimation.
5. Protocolized age estimation studies based on color changes are recommended.

## Data Availability

The datasets used and/or analyzed during the current study are available from the corresponding authors upon reasonable request.

## Appendix 1

**Table:**

Author	Device	Setting Devices	Measure Conditions	Analysis System	Region of measure
Cho et al. [44]	Tristimulus colorimeter (CM: Chroma Meter CR 321, Minolta, Osaka,Japan) Shade Vision System (SV: X-rite, Grandville, MO, USA)	CM: The aperture diameter of the colorimeter:3mm 45◦ illumination/0◦ viewing angle SV	Calibration using a white standard tile aperture No direct sunlight Measurements repeated three times	Portable device and measure condition	In contact with the center of the tooth
da Silva et al. [42]	Sony cybershot DSC (Digital Still Camera) camera (Sony USA – Sony Corporation of America)	Macro f/ 2.8, ISO 400 Speed of aperture 30 No flash	Frankfurt plane Distance of 65 cm from the camera. Fixed camera was used. Dark environment with UV lamp (only light source)	Portable device and measure condition Software ScanWhite DMC/	Vestibular surface No contact
Demirel and Tuncdemir [5]	Vita Easyshade spectrophotometer (Vita Easyshade V; Vita Zahnfabrik)	D-65 illumination	Artificial light conditions	Portable device and measure condition	In contact with the middle third of the labial tooth surface
Devos et al. [40]	ShadeEye-NCC™ Dental Chroma Meter (Shofu, San Marcos, CA, USA)		Against a black background	Portable device	Ex vivo: Vestibular aspect of the tooth crown (CV) as well as on the mesial (MR) and vestibular (VR) surface of the root. In contact with the enamel or dentine surface at a vertical level of at least 4 mm away from the vestibular cementum-enamel junction. In vivo: In contact with vestibular enamel, at least 2mm coronal to the gingival border
Eiffler et al. [33]	VITA Easyshade 1 spectrophotometer (VITA Zahnfabrik, Bad Säckingen, Germany)	Software release 11R(b) D-65 illumination With 2° observer	Environmental conditions were not standardized. A single measurement	Portable device and measure condition	In contact with the middle third of labial tooth surface (2 mm from gingival margin and incisal edge)
Gómez Polo et al. [29]	Vita Easyshade Compact spectrophotometer (Vita-Zahnfabrik, Bad Sackingen, Germany)	-	The color of the surroundings was neutralized with a grey cloth. Standardized lighting conditions: D65 daylight fluorescent tubes with a light intensity of 1,500 lux.	Portable device and measure condition “Single Tooth” Mode	In contact with the middle third of the labial tooth surface
Gozalo-Diaz et al. [43]	Spectroradiometer (PR 705; Photo Research Inc)	Spectroradiometer and the optic light cable, positioned at a 45-degree angle inferior to the horizontal plane. Optical configuration of 0-degree observation and 45-degree illumination to the object. CIE Standard Human (2-degree) Observer. D65 illumination	The subjects were seated with their lower jaw and forehead resting lightly on a head-frame Xenon arc lamp (300W; Oriel Instruments) connected via fiber optic light cable Distance of 8 cm from the measured object with a measurement aperture size of 1 mm in diameter	Not portable Spectral reflectance obtained from 380 nm to 780 nm with a 2-nm interval (SpectraWin 2; Photo Research, Inc)	Center of labial surface No contact
Greta et al. [39]	Vita Easyshade Advance spectrophotometer (Vita-Zahnfabrik, Bad Sackingen, Germany)	-	Artificial illuminants (ceiling fluorescent tube lighting OSRAM LumiluxDeluxe 36W/965 5500K) Measurements repeated three times	Portable device and measure condition “Single Tooth” Mode	Contact held at 90° to the tooth surface, in its middle third
Haddad et al. [34]	VITA Easyshade 1 spectrophotometer (VITA Zahnfabrik, Bad Säckingen, Germany)	-	-	Portable device and measure condition	In contact with the middle third of the clinical crown at 90º to the labial surface
Haralur [18]	Vita Easyshade spectrophotometer (VITA Zahnfabrik GmbH, Bad Sackingen, Germany)	-	Any light condition Average shade from both maxillary central incisors	Portable device and measure condition	In contact with the middle portion of the tooth and perpendicular to tooth surface
Hassel et al. [35]	VITA Easyshade 1 spectrophotometer (Easyshade 1 (ES), serial no. 503006; Vita Zahnfabrik, Bad Säckingen, Germany)	-	-	Portable device and measure condition	In contact with the middle third of the tooth (2 mm from the incisal and cervical edges)
Karaman et al. [36]	VITA Easyshade V spectrophotometer (VITA Zahnfabrik)	-	The measurements were conducted in daylight and clear weather, between 10:00 am and 12:00 pm. Any bright colors on the patients’ faces or clothing were covered A grey background color was used to prevent background reflection Tooth shade average: three measurements were performed	Portable device and measure condition “L, C, and H” data conversions to the L*, a*, and b* values were carried out using those found on the EasyRGB website	In contact with the middle third of the labial surface
Kim [37]	VITA Easyshade V, spectrophotometer (VITA Zahnfabrik; Bad Säckingen; Germany)	Software version V507h With 2º observer D65 LED light source	-	Portable device and measure condition “Base shade estimation” mode	In contact with the central tooth area and perpendicular to the tooth surface
Krasniqi et al. [41]	VITA Easyshade spectrophotometer	-	-	Portalbe device and measure condition Program “Tooth Areas” of the spectrophotometer	In contact with the cervical, middle and incisal areas of the tooth
Martin-de las Heras et al. [31]	PR-704/pc SpectraScan spectroradiometer.	Standard illuminant (D65)	Standard conditions: special chamber (model CAC 120 of Verivide), white standard as a reference	Not portable	Root dentine Non contact
Martin-de-las-Heras et al. [32]	Spectro-color device (Dr Lange, Keison Products Co, England)	D65 for the illuminant conditions 8° for the standard observer	-	Portable device “Analysis mode”	In contact with the vestibular surface of the teeth
Rubiño et al. [30]	TOPCON BM-5 colorimeter	-	Diffuse illumination (white walls and fluorescent lights)	Portable device and measure condition	Central portion of the tooth
Wee et al. [38]	Spectroradiometer (PR 705; Photo Research Inc) and Xenon arc lamp (300W; Oriel Instruments) connected via fiber optic light cable	Spectroradiometer and the optic light cable, positioned at a 45-degree angle inferior to the horizontal plane Optical configuration of 0-degree observation and 45-degree illumination to the object CIE Standard Human (2-degree) observer at an infinite enamel thickness for each participant D65 illumination	Participants were seated with their mandible and forehead resting on a head frame, and cheek retractors Distance of 9 cm from the measured object with a measurement aperture size of 1 mm in diameter	Not portable	Central one-ninth of these incisors No contact
